# Supplementary material for: A Non-Gradual Development Process of Cicada Eyes at the End of the Fifth-Instar Nymphal Stage to Obtain Visual Ability
Source: Insects. 2022 Dec 16;13(12):1170. doi: 10.3390/insects13121170 (PMC9787698; doi:10.3390/insects13121170)
Supplement: Supplementary file 1 [file insects-13-01170-s001.zip › Supplementary information.pdf]

Supplementary Information

Title: A non-gradual development process of cicada eyes occurred at the end of the fifth-instar nymphal stage to obtain visual ability

Authors: Min-Jing Su[1], Fei-Min Yuan[1], Tian-Tian Li[1], Cong Wei[1,*]

[1] Key Laboratory of Plant Protection Resources and Pest Management of the Ministry of Education, College of Plant Protection, Northwest A&F University, Yangling, Shaanxi 712100, China

Correspondence: Cong Wei: congwei@nwsuaf.edu.cn

Supplementary figure captions

Figure S1. Cluster analyses based on all identified differentially expressed genes (DEGs). (**A**) Unsupervised hierarchical clustering of all DEGs in transcriptomes of compound eyes at five developmental stages. The numbers 1, 2 and 3 at the end of the sample represent three biological replicates. (**B**) Fuzzy c-means clustering identified ten distinct temporal patterns of expression of all DEGs. The x axis represents five developmental stages, while the y axis represents log2-transformed, normalized intensity ratios in each stage. N5R, the 5th-instar nymph with white compound eyes. N5R, the 5th-instar nymph with red compound eyes. N5B, the 5th-instar nymph with dark brown compound eye. A3, the adults after emergence 3 h. A48, the adults after emergence 48 h.

Figure S2. Functional enrichment analysis of stage-specific DEGs. GO function and KEGG pathway enrichment analyses were performed on the stage-specific DEGs. The gene sets with p-value < 0.01 and Enrichment Fold > 5 are showed and mainly related to coloration, phototransduction, hormonal regulation, innate immune response and cuticle development. N5R, the 5th-instar nymph with white compound eyes. N5R, the 5th-instar nymph with red compound eyes. N5B, the 5th-instar nymph with dark brown compound eye. A3, the adults after emergence 3 h. A48, the adults after emergence 48 h.

Figure S3. Phylogenetic analysis of four opsin genes in *M. mongolica*. The phylogenetic tree was based on Neighbor-Joining (NJ) method. Different opsin nucleotide sequences are grouped into four clusters, which belonged to three orders and an outgroup, and are assigned

with different colors. Numbers at nodes indicate the bootstrap values. The 29 sequences are indicated by the corresponding scientific names of species followed by their gene names and accession numbers. The sequences of four opsins studied in our study are indicated by solid red rectangular boxs.

Figure S4. Alignment of protein sequences of 29 opsins. Four opsins (MmLop1, MmLop2, MmBLop1 and MmUVop1) are from *M. mongolica*. The seven transmembrane domains are marked above the alignments as TMD1–7.

Figure S5. Expression patterns of key DEGs related to pathways of innate immune activation and regulation. There were 39 immune-related key genes expressed differently in the eyes from N5W to A48, which were classified into three functional groups, including pattern recognition proteins, signal transduction molecules and effector proteins. The 19 pattern-recognition protein genes were assorted into one *GNBP1*, one *PGRP-L*, three *PGRP-Ss*, and 14 *βGRPs*. Signal transduction molecules involved the genes encoding Spätzle-processing enzyme (SPE), Spätzle (Spz, four genes), Toll (three genes), Tollip, and Relish. Immune-responsive effectors involved the genes encoding prophenoloxidases (PPO, five genes) and antimicrobial peptides (AMPs, five genes). The heatmaps based on TPM show the expression levels of the corresponding protein or enzyme genes in five development stages (from left to right, the 5th-instar with white compound eyes (N5W), with red compound eyes (N5R), with dark brown compound eye (N5B), the adults after emergence 3 h (A3), 48 h (A48)).

Figure S6. Expression patterns of key DEGs involved in chitin-related pathways and cuticular proteins. There were 62 cuticular protein (CP) genes expressed differently from N5W to A48, including 39, 8, 8, 5 and 2 genes in the RR-2, RR-1, CPAP1, CPAP3 and Tweedle families, respectively. The chitin-related genes, including one chitin synthetase (CHS), one chitin deacetylase (CDA) and eight chitinase (Cht) genes, were expressed dynamically during the five development stages. The heatmaps based on TPM show the expression levels of the corresponding protein or enzyme genes in five development stages (from left to right, the 5th-instar with white compound eyes (N5W), with red compound eyes (N5R), with dark brown compound eye (N5B), the adults after emergence 3 h (A3), 48 h (A48)).

Figure S7. Gene expression levels of differentially expressed genes through RNA-seq and RT-qPCR analyses. N5R, the 5th-instar nymph with white compound eyes. N5R, the 5th-instar nymph with red compound eyes. N5B, the 5th-instar nymph with dark brown compound eye. A3, the adults after emergence 3 h. A48, the adults after emergence 48 h.

Supplementary table captions

Table S1. Names and sequences of opsin genes in *M. mongolica*.

Table S2. Specific primer pairs used in RT-qPCR.

Table S3. DEGs related to the pathways of innate immune activation and regulation.

Corresponding to Figure. S5.

Table S4. DEGs related to cuticle development. Corresponding to Figure. S6.
