# Supplementary figures and images for: A Non-Gradual Development Process of Cicada Eyes at the End of the Fifth-Instar Nymphal Stage to Obtain Visual Ability
Source: Insects. 2022 Dec 16;13(12):1170. doi: 10.3390/insects13121170 (PMC9787698; doi:10.3390/insects13121170)

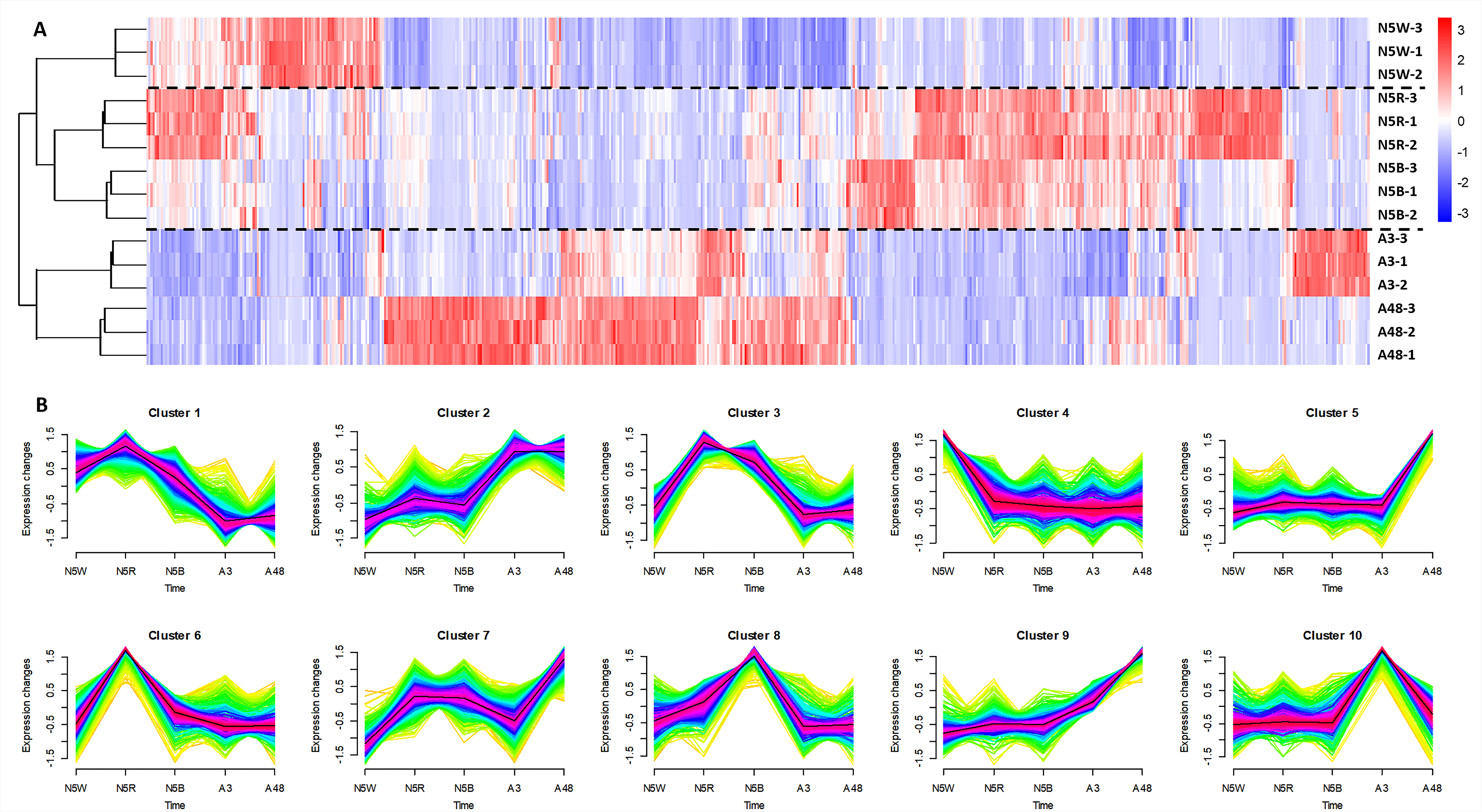

Supplement: Supplementary file 1 [file insects-13-01170-s001.zip › Figure S1.tif]

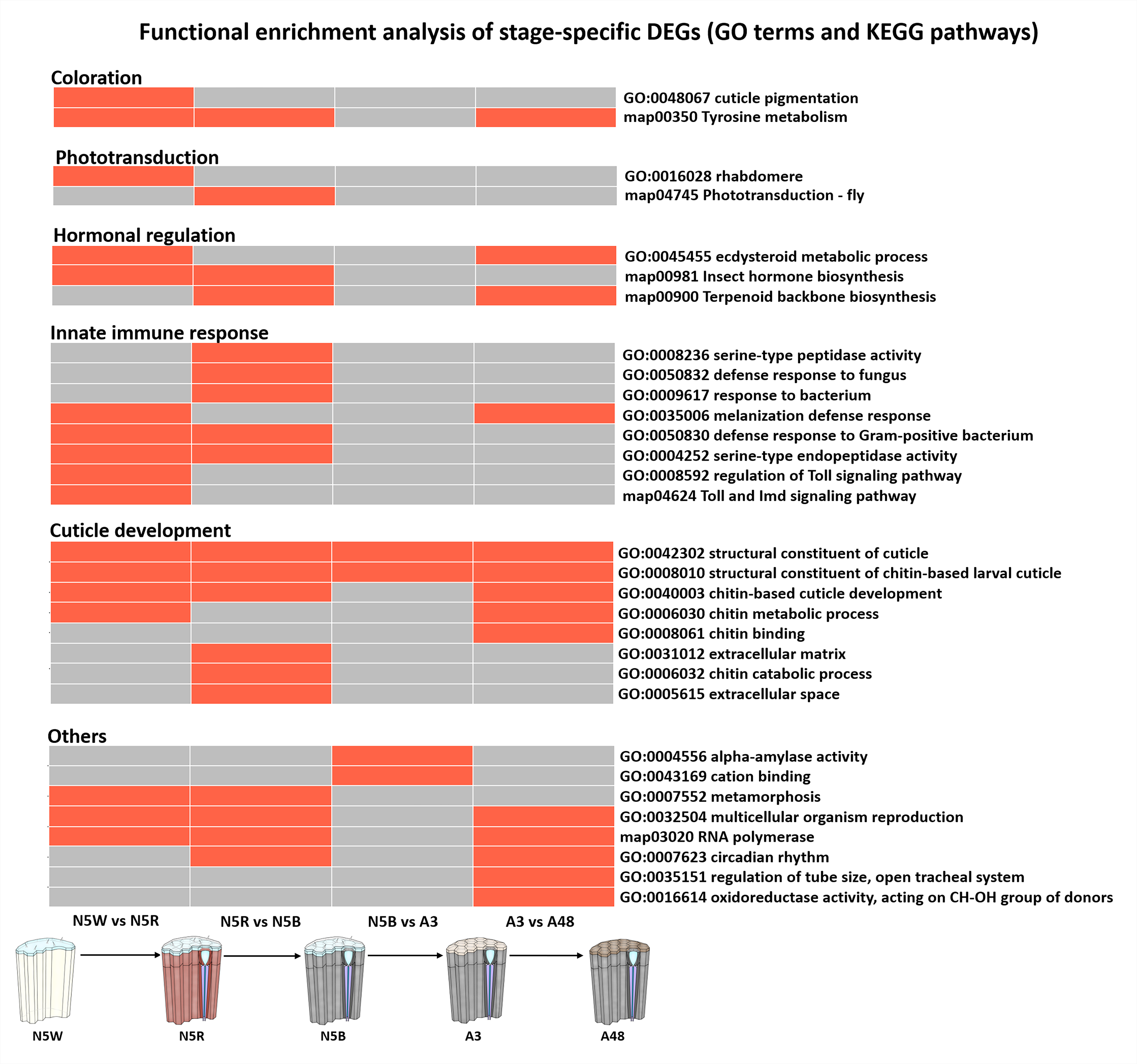

Supplement: Supplementary file 1 [file insects-13-01170-s001.zip › Figure S2.tif]

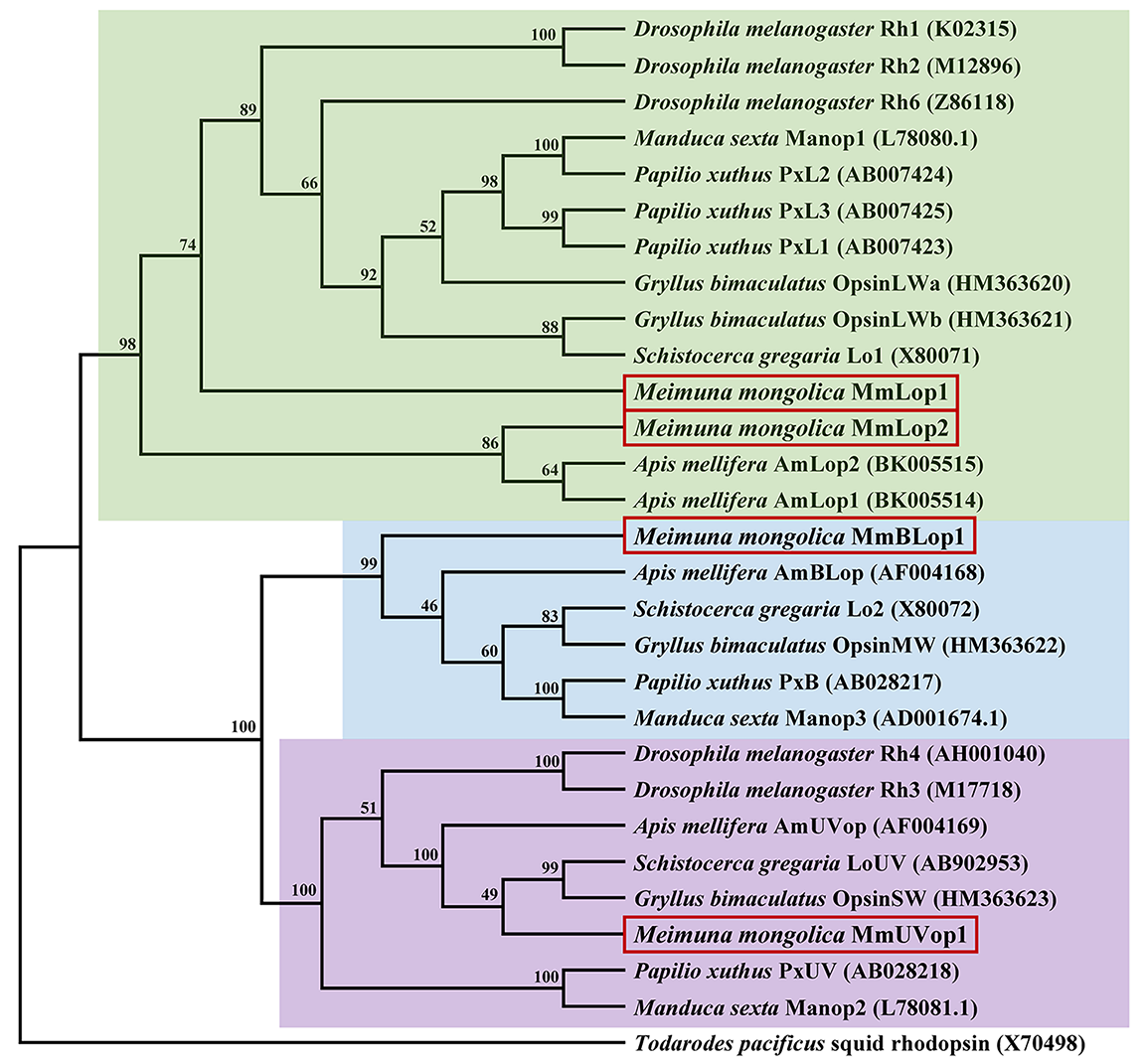

Supplement: Supplementary file 1 [file insects-13-01170-s001.zip › Figure S3.tif]

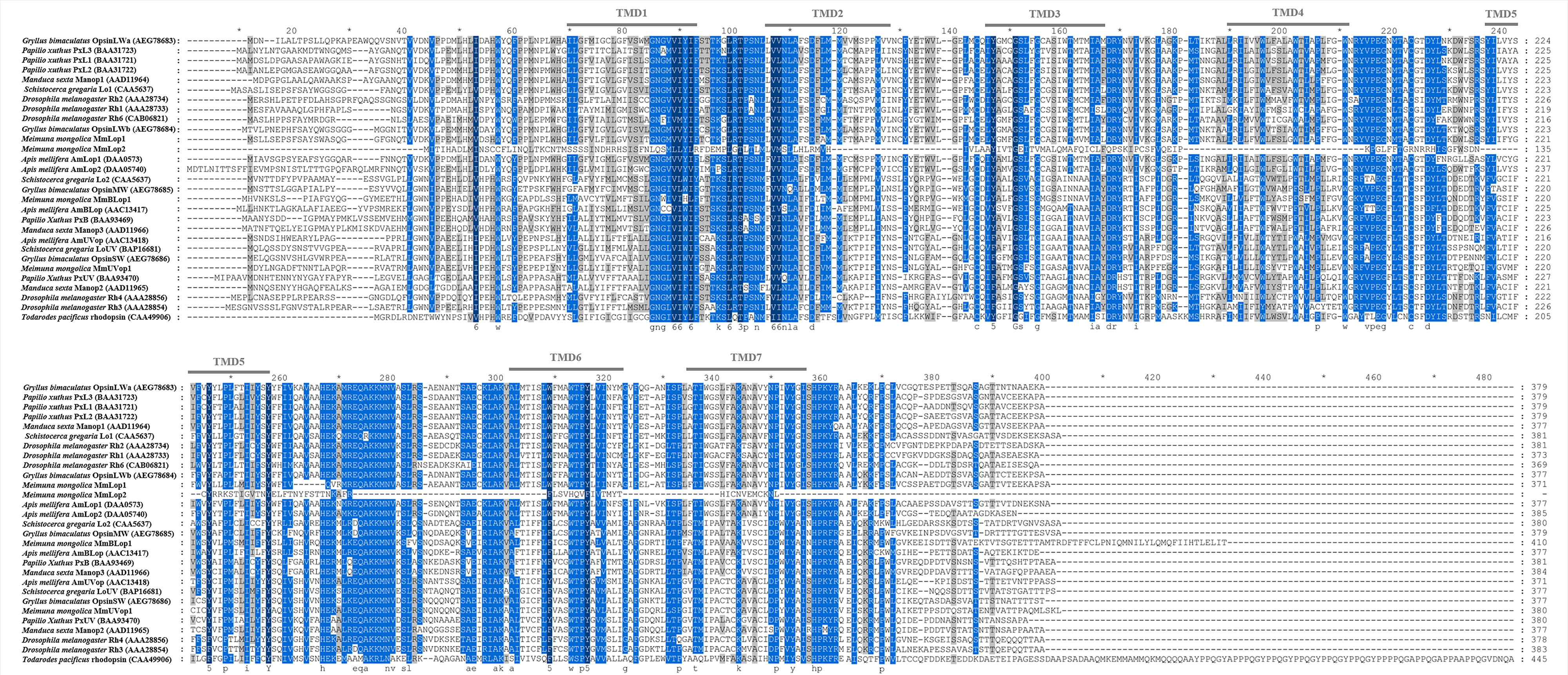

Supplement: Supplementary file 1 [file insects-13-01170-s001.zip › Figure S4.tif]

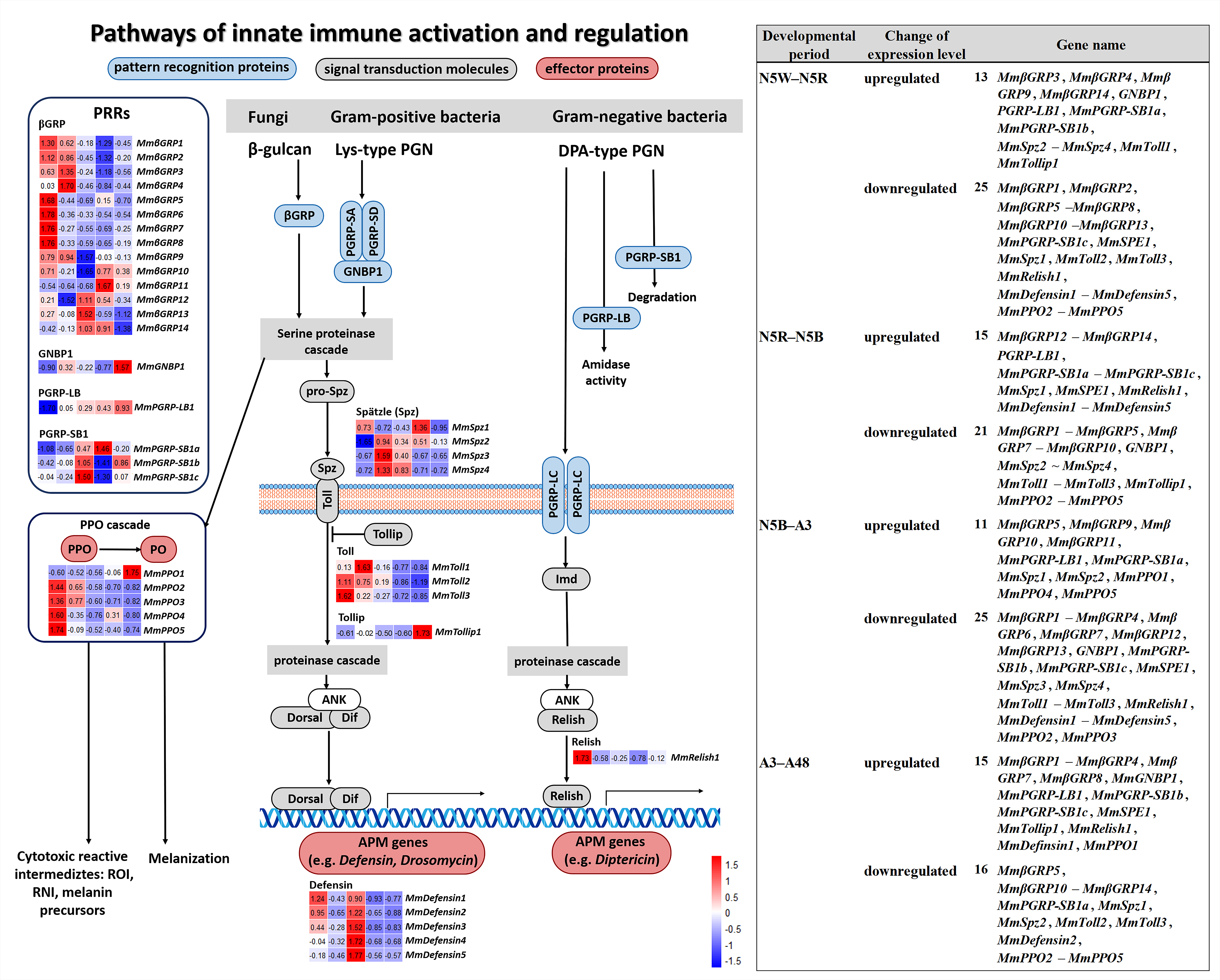

Supplement: Supplementary file 1 [file insects-13-01170-s001.zip › Figure S5.tif]

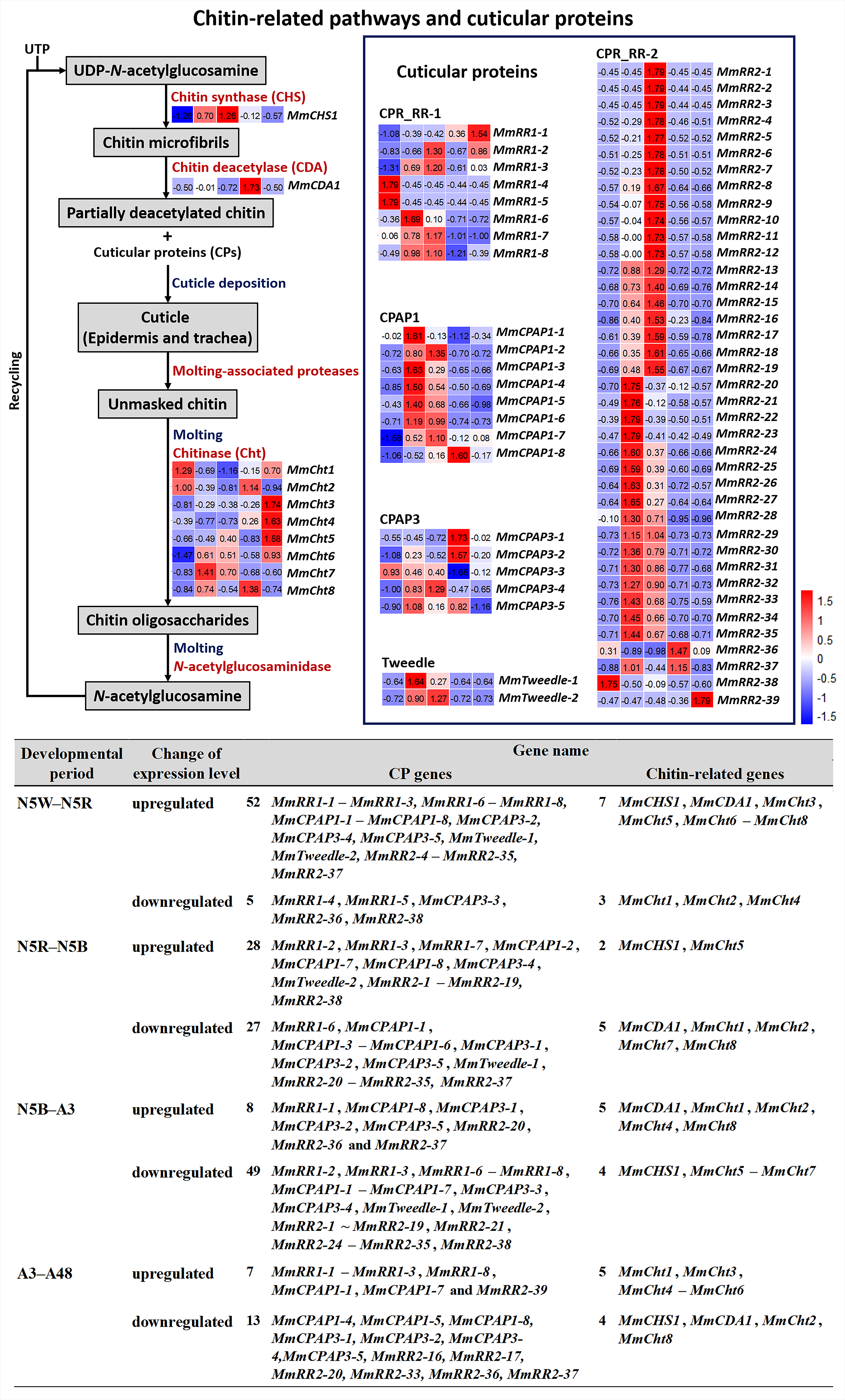

Supplement: Supplementary file 1 [file insects-13-01170-s001.zip › Figure S6.tif]

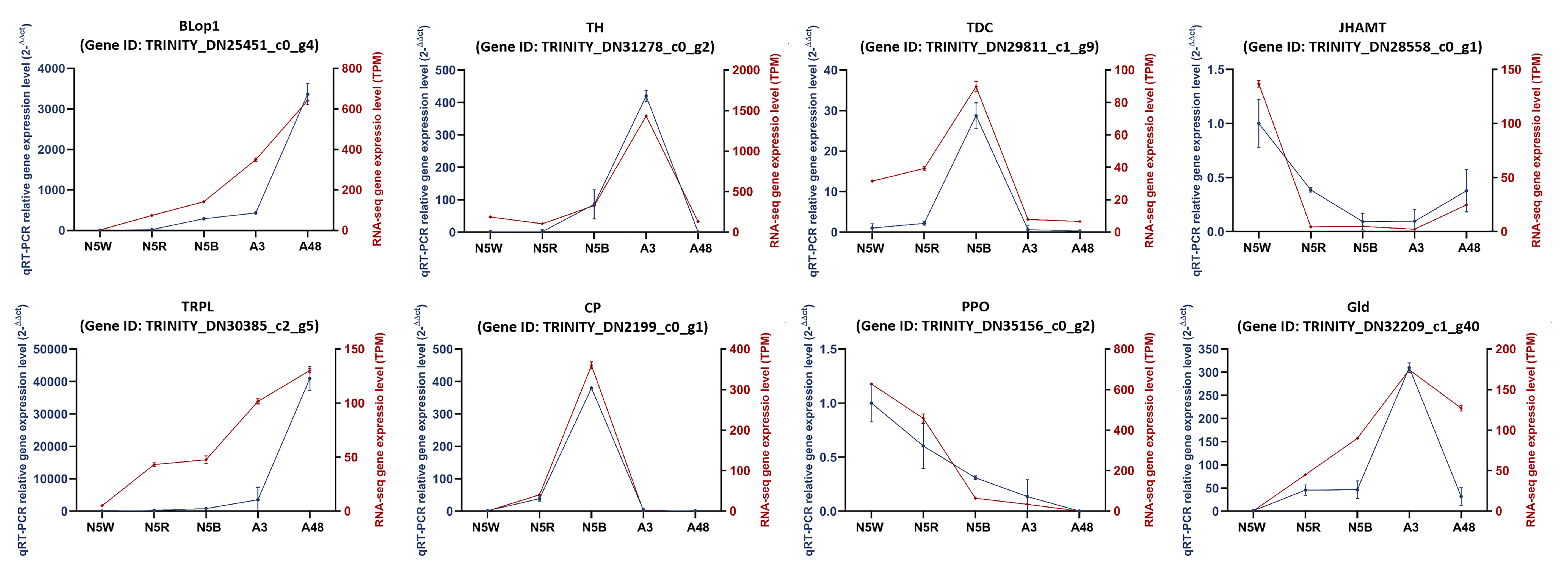

Supplement: Supplementary file 1 [file insects-13-01170-s001.zip › Figure S7.tif]
